# Multiple Routes of Antibody-Dependent Enhancement of SARS-CoV-2 Infection

**DOI:** 10.1128/spectrum.01553-21

**Published:** 2022-03-23

**Authors:** Kosuke Okuya, Takanari Hattori, Takeshi Saito, Yoshihiro Takadate, Michihito Sasaki, Wakako Furuyama, Andrea Marzi, Yoichi Ohiro, Satoshi Konno, Takeshi Hattori, Ayato Takada

**Affiliations:** a Division of Global Epidemiology, International Institute for Zoonosis Control, Hokkaido University grid.39158.36grid.412167.7 , Sapporo, Japan; b Division of Molecular Pathobiology, International Institute for Zoonosis Control, Hokkaido University grid.39158.36grid.412167.7 , Sapporo, Japan; c Laboratory of Virology, Division of Intramural Research, National Institute of Allergy and Infectious Diseases, National Institutes of Health, Rocky Mountain Laboratories, Hamilton, Montana, USA; d Department of Oral and Maxillofacial Surgery, Hokkaido University grid.39158.36grid.412167.7 Graduate School of Dental Medicine, Sapporo, Japan; e Department of Respiratory Medicine, Faculty of Medicine and Graduate School of Medicine, Hokkaido University grid.39158.36grid.412167.7 , Sapporo, Japan; f Department of Respiratory Medicine, National Hospital Organization Hokkaido Medical Center, Sapporo, Japan; g International Collaboration Unit, International Institute for Zoonosis Control, Hokkaido University grid.39158.36grid.412167.7 , Sapporo, Japan; Pontificia Universidad Católica de Chile

**Keywords:** ADE, antibody-dependent enhancement, C1q, COVID-19, complement, Fc receptor, FcR, SARS-CoV-2

## Abstract

Antibody-dependent enhancement (ADE) of infection is generally known for many viruses. A potential risk of ADE in severe acute respiratory syndrome coronavirus 2 (SARS-CoV-2) infection has also been discussed since the beginning of the coronavirus disease 2019 (COVID-19) pandemic; however, clinical evidence of the presence of antibodies with ADE potential is limited. Here, we show that ADE antibodies are produced by SARS-CoV-2 infection and the ADE process can be mediated by at least two different host factors, Fcγ receptor (FcγR) and complement component C1q. Of 89 serum samples collected from acute or convalescent COVID-19 patients, 62.9% were found to be positive for SARS-CoV-2-specific IgG. FcγR- and/or C1q-mediated ADE were detected in 50% of the IgG-positive sera, whereas most of them showed neutralizing activity in the absence of FcγR and C1q. Importantly, ADE antibodies were found in 41.4% of the acute COVID-19 patients. Neutralizing activity was also detected in most of the IgG-positive sera, but it was counteracted by ADE in subneutralizing conditions in the presence of FcγR or C1q. Although the clinical importance of ADE needs to be further investigated with larger numbers of COVID-19 patient samples, our data suggest that SARS-CoV-2 utilizes multiple mechanisms of ADE. C1q-mediated ADE may particularly have a clinical impact since C1q is present at high concentrations in plasma and its receptors are ubiquitously expressed on the surfaces of many types of cells, including respiratory epithelial cells, which SARS-CoV-2 primarily infects.

**IMPORTANCE** Potential risks of antibody-dependent enhancement (ADE) in the coronavirus disease 2019 (COVID-19) caused by severe acute respiratory syndrome coronavirus 2 (SARS-CoV-2) infection has been discussed and the proposed mechanism mostly depends on the Fc gamma receptor (FcγR). However, since FcγRs are exclusively expressed on immune cells, which are not primary targets of SARS-CoV-2, the clinical importance of ADE of SARS-CoV-2 infection remains controversial. Our study demonstrates that SARS-CoV-2 infection induces antibodies that increase SARS-CoV-2 infection through another ADE mechanism in which complement component C1q mediates the enhancement. Although neutralizing activity was also detected in the serum samples, it was counteracted by ADE in the presence of FcγR or C1q. Considering the ubiquity of C1q and its cellular receptors, C1q-mediated ADE may more likely occur in respiratory epithelial cells, which SARS-CoV-2 primarily infects. Our data highlight the importance of careful monitoring of the antibody properties in COVID-19 convalescent and vaccinated individuals.

## INTRODUCTION

Coronaviruses (CoVs), order *Nidovirales*, family *Coronaviridae*, are enveloped positive-sense single-stranded RNA viruses, including a number of pathogens that infect avian and mammalian species, including humans. Severe acute respiratory syndrome coronavirus 2 (SARS-CoV-2), the causative agent of coronavirus disease 2019 (COVID-19), has been associated with 222,406,582 cases and 4,592,934 deaths globally as of September 9th, 2021 (WHO Coronavirus (COVID-19) Dashboard | WHO Coronavirus (COVID-19) Dashboard With Vaccination Data). The increasing number of COVID-19 patients in the pandemic situation has enormously damaged the world economy as well as global public health.

CoVs have an envelope spike protein (S) that mediates viral entry into cells and this is one of the key determinants of host and tissue tropisms (1). It has been well documented that SARS-CoV-2 uses angiotensin-converting enzyme receptor 2 (ACE2) as a receptor on the cell surface and that antibodies specific to the SARS-CoV-2 S inhibit its binding to ACE2 (2, 3). Previous studies have demonstrated that neutralizing antibodies induced in COVID-19 patients are sustained up to 9 months with an initial decay decelerating after a few weeks (4–7). However, it has also been suggested that S-specific antibodies may mediate antibody-dependent enhancement (ADE) of SARS-CoV-2 infection (8, 9). ADE is a phenomenon in which virus-specific antibodies increase infection and has been reported for many viruses *in vitro* (e.g., dengue virus, West Nile virus, human immunodeficiency virus (HIV), Ebola virus (EBOV), and feline infectious peritonitis virus) (10, 11). Although ADE is often associated with increased severity and/or imunopathology of viral diseases (8, 12, 13), it has been mostly studied for *in vitro* infection focusing on increased viral entry into target cells and most studies until now do not show that ADE will be a clinical problem for the disease caused by SARS-CoV-2.

Previous studies have demonstrated that monoclonal antibodies (MAbs) recognizing a particular epitope on the S of SARS-CoV induce ADE and the presence of these antibodies during the infection results in increased virus replication and exacerbation of disease in nonhuman primates (13). Since SARS-CoV-2 shares some epitopes in the S with SARS-CoV (14, 15) and ADE antibodies are also reported for other CoVs such as Middle East respiratory syndrome coronavirus (MERS-CoV) and feline infectious peritonitis virus (16, 17), the potential risk of ADE has been discussed for SARS-CoV-2 infection. It is well known that ADE mostly depends on the cross-linking of virus-antibody complexes through interaction with the fragment crystallizable (Fc) portion of an antibody to Fcγ receptors (FcγRs) expressed on immune cells. Accordingly, FcγR-mediated ADE of SARS-CoV and MERS-CoV infections has been previously reported (17, 18). Recently, some human MAbs specific to the S of SARS-CoV-2 were found to enhance the virus infection of human B lymphoblastoid and chronic myelogenous leukemia cell lines *in vitro* through the FcγR-mediated pathway (19, 20). It has also been shown that ADE antibodies most likely recognize epitopes on the receptor binding site and N-terminal domain of the S although detailed information on the epitopes involved in ADE is still limited (19). However, another study reported that serum samples from convalescent COVID-19 patients did not induce ADE of SARS-CoV-2 entry into human monocyte-derived macrophages (21). Importantly, FcγRs are expressed exclusively in immune cells such as macrophages, B cells, and natural killer cells, which are not the principal targets of SARS-CoV, MERS-CoV, and SARS-CoV-2, raising doubt as to the clinical importance of ADE during SARS-CoV-2 infection (8, 22).

In this study, we investigated two different mechanisms of ADE of SARS-CoV-2 entry into cells, mediated by FcγR and complement component C1q. C1q-mediated ADE is a mechanism independent from FcγRs that potentially enhances viral infection of many types of cells lacking FcγRs (11, 23, 24). Using serum samples of acute and convalescent COVID-19 patients, we analyzed their neutralizing, FcγR-, and C1q-mediated ADE activities *in vitro* and found evidence of both types of ADE that counteracted neutralizing activities depending on the concentrations of antibodies.

## RESULTS

### SARS-CoV-2-specfic IgG and IgM detected in the serum samples.

**FIG 1:**
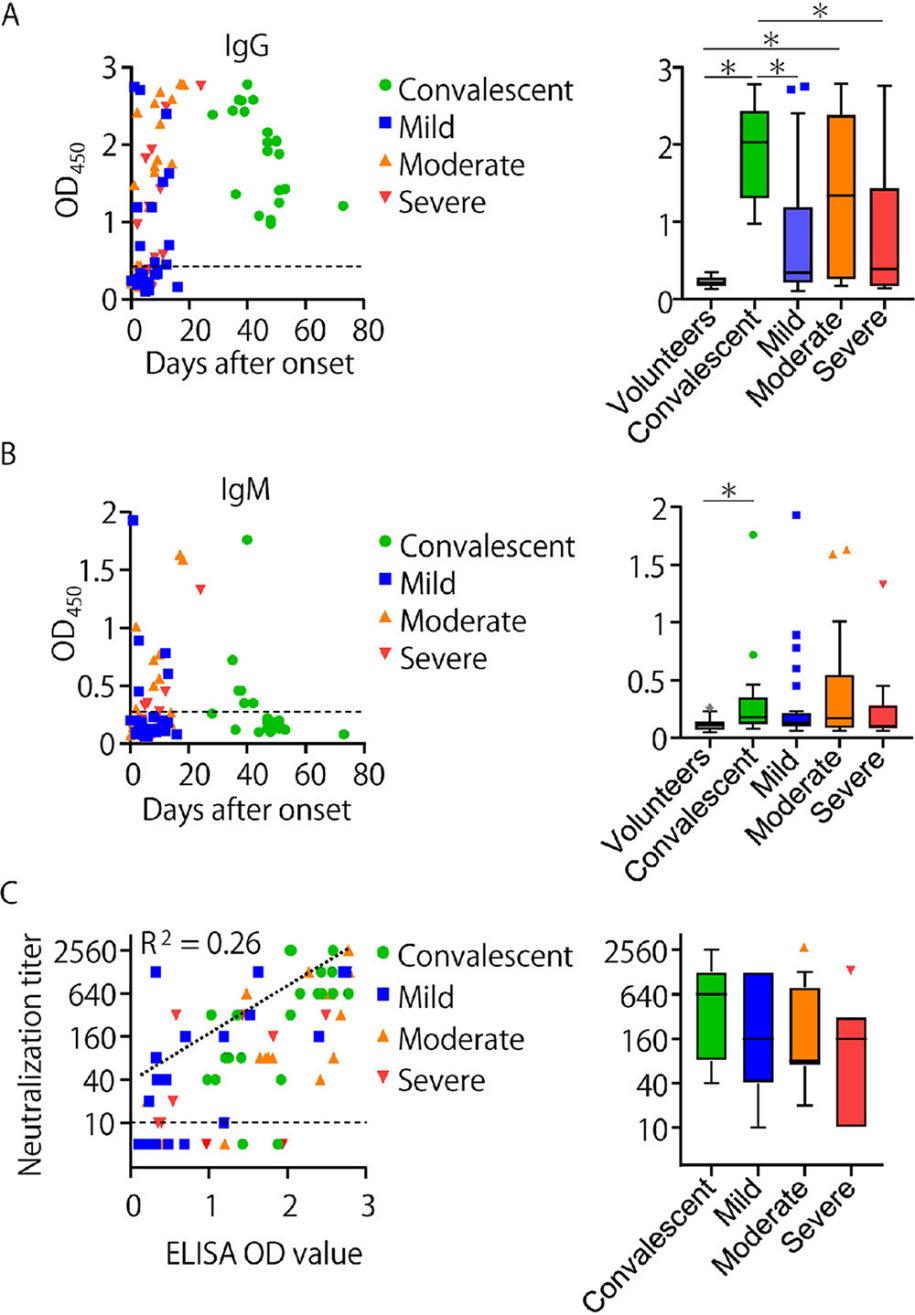
SARS-CoV-2-specific IgG, IgM, and neutralizing antibodies detected in serum samples collected from COVID-19 acute and convalescent patients. (A) IgG and (B) IgM antibodies reactive to the SARS-CoV-2 whole virus antigens were measured in ELISA. (A, B) The cutoff values (dashed lines) were determined as averages ± 3×SD of the OD values of healthy volunteers. (C) Neutralizing titers and ELISA OD values of the samples are plotted with a regression line (dotted line). The detection limit of neutralizing titers was 10 (reciprocal dilution) as shown by the dashed line. Samples below the limit of detection are not shown (C, right panel). Each box with a horizontal black line represents the IQR and median. Symbols represent outlying plots located over 1.5 × IQR from the upper quartile. Whiskers are shown from the highest and lowest values within a fence to the 3rd and 1st quartiles, respectively. Asterisks indicate significant differences (*P* < 0.05) determined using the Kruskal-Wallis test followed by Dunn’s multiple-comparison test.

Serum samples were obtained from 89 laboratory-confirmed COVID-19 cases (21 convalescent and 68 acute patients) and 23 healthy volunteers. Days from the onset of the disease to blood sampling were 0–24 (acute) and 28–73 (convalescent). The serum samples of acute COVID-19 patients were divided into three groups based on the severity of symptoms; mild (*n* = 25; without need of oxygen administration), moderate (*n* = 24; requiring oxygen administration), and severe (*n* = 19; requiring mechanical ventilation). IgG and IgM antibodies specific to SARS-CoV-2 proteins were measured by enzyme-linked immunosorbent assay (ELISA) (Fig. 1A and B, and Table 1). As expected, virus-specific IgG antibodies were detected in all of the serum samples collected from convalescents, whereas only 6 (28.6%) of the samples were IgM-positive. Virus-specific IgG antibodies were also detected in 44%, 62.5%, and 47.3% of the acute patients with mild, moderate, and severe symptoms, respectively. The antibody levels estimated by ELISA optical density (OD) values of patients with mild and severe symptoms were significantly lower than those of the convalescents (Fig. 1A right panel). Overall, SARS-CoV-2-specific IgG antibodies were detected in 62.9% (56/89) of the convalescents and patients. Although IgM antibodies were also detected in the patients, the positive rates were lower than for IgG and a significant difference was only found between the healthy volunteers and convalescents (Fig. 1B). Notably, IgG and IgM antibodies reacting to the SARS-CoV-2 antigen were detectable even a few days after the onset of the disease in some of the patients (Fig. 1A and B).

**TABLE 1:** Presence of anti-SARS-CoV-2 antibodies in COVID-19 acute and convalescent patients

Group	IgG	IgM	Neutralization
Healthy volunteer	0% (0/23)	0% (0/23)	0% (0/23)
Convalescent	100% (21/21)	28.6% (6/21)	90.5% (19/21)
Mild disease	44% (11/25)	20% (5/25)	56% (14/25)
Moderate disease	62.5% (15/24)	33.3% (8/24)	58.3% (14/24)
Severe disease	47.3% (9/19)	21.1% (4/19)	47.4% (9/19)

### Neutralizing activity of the serum samples against the vesicular stomatitis virus (VSV) pseudotyped with SARS CoV-2 S (VSV-SARS2).

We then analyzed neutralizing antibodies in the serum samples (Fig. 1C). The neutralization titer of each serum sample was determined using VSV-SARS2, which enabled us to specifically investigate biological activities of antibodies specific to the S (25–27). We found that neutralization titers (the highest serum dilution that gave 50% inhibition of infected cells) were positively correlated with the OD values given by IgG reactivity in ELISA (R^2^ = 0.26) (Fig. 1C, left panel). In addition, most of the convalescent-phase sera (19/21) were found to have neutralizing antibodies with titers ranging from 20 to 2560. On the other hand, appreciable neutralization activity (i.e., titers of >10) was detected in 56%, 58.3%, and 47.4% of the patients with mild, moderate, and severe symptoms, respectively (Table 1). There was no significant difference in neutralizing titers among the groups (Fig. 1C, right panel).

### FcγR- and C1q-mediated ADE activities of the serum samples.

**FIG 2:**
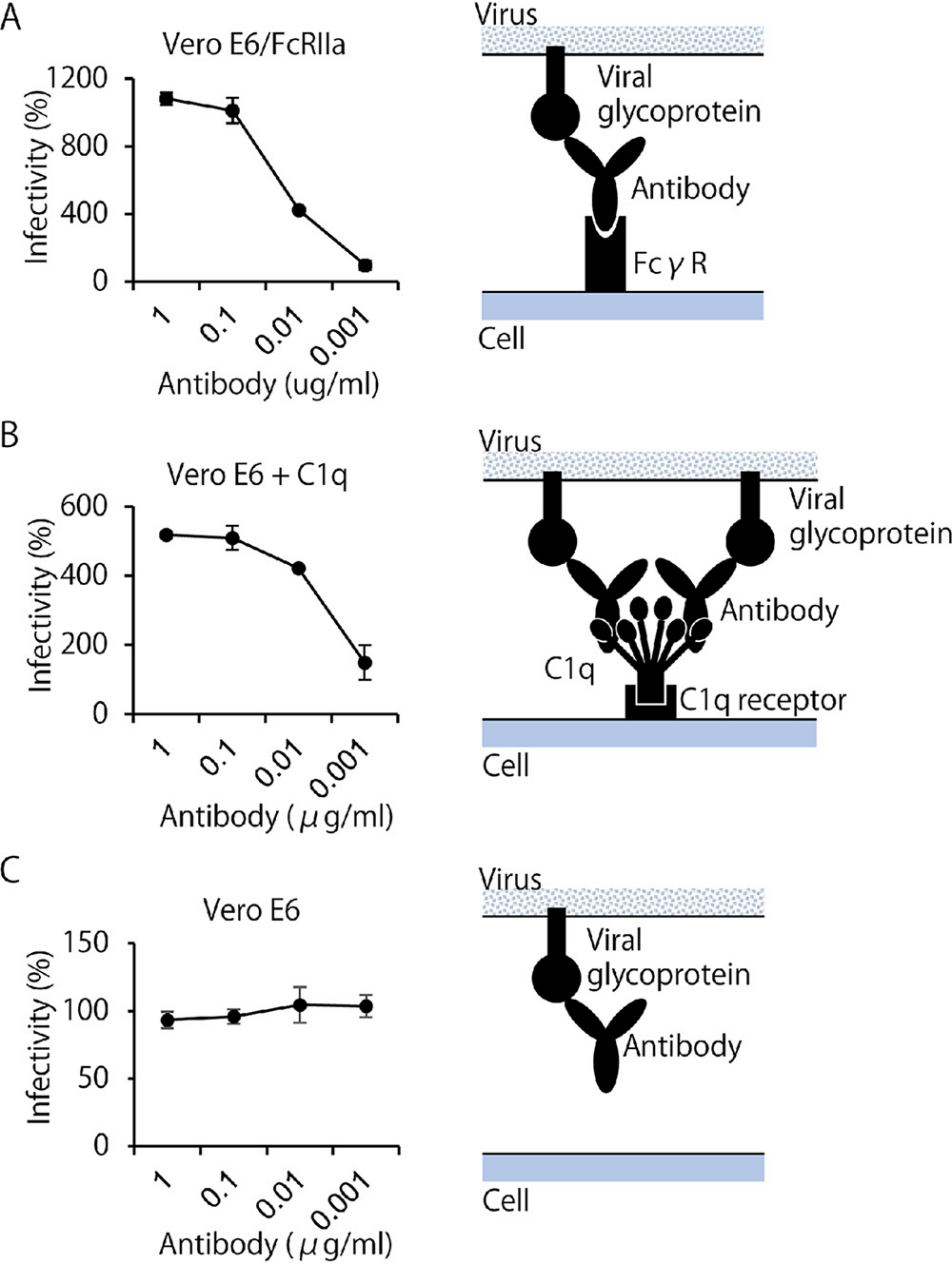
ADE mechanisms and assay validation for FcγR- and C1q-mediated ADE using VSV-EBOV and an EBOV glycoprotein-specific monoclonal antibody. Infectious titers of VSV-EBOV mixed with indicated concentrations of monoclonal antibody ZGP12/1.1 (27) were measured in (A) Vero E6/FcγRIIa cells, (B) Vero E6 cells in the presence of C1q, and (C) Vero E6 cells in the absence of C1q. The relative numbers of infected cells were calculated by setting the number of GFP-positive cells in the absence of the antibody to 100%. Dots and error bars indicate the means and standard deviations of triplicate wells, respectively. Right panels show schematics of the respective conditions.

**FIG 3:**
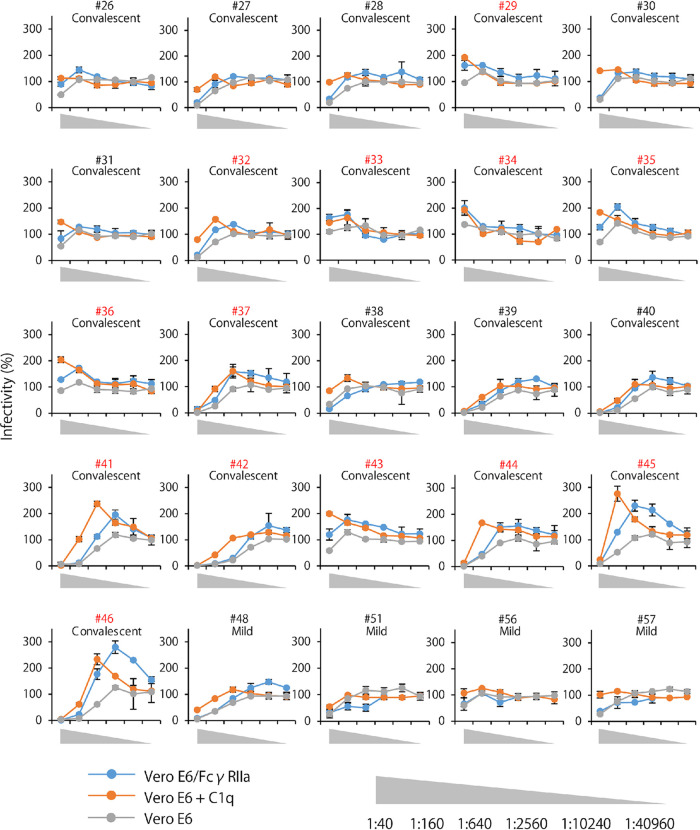

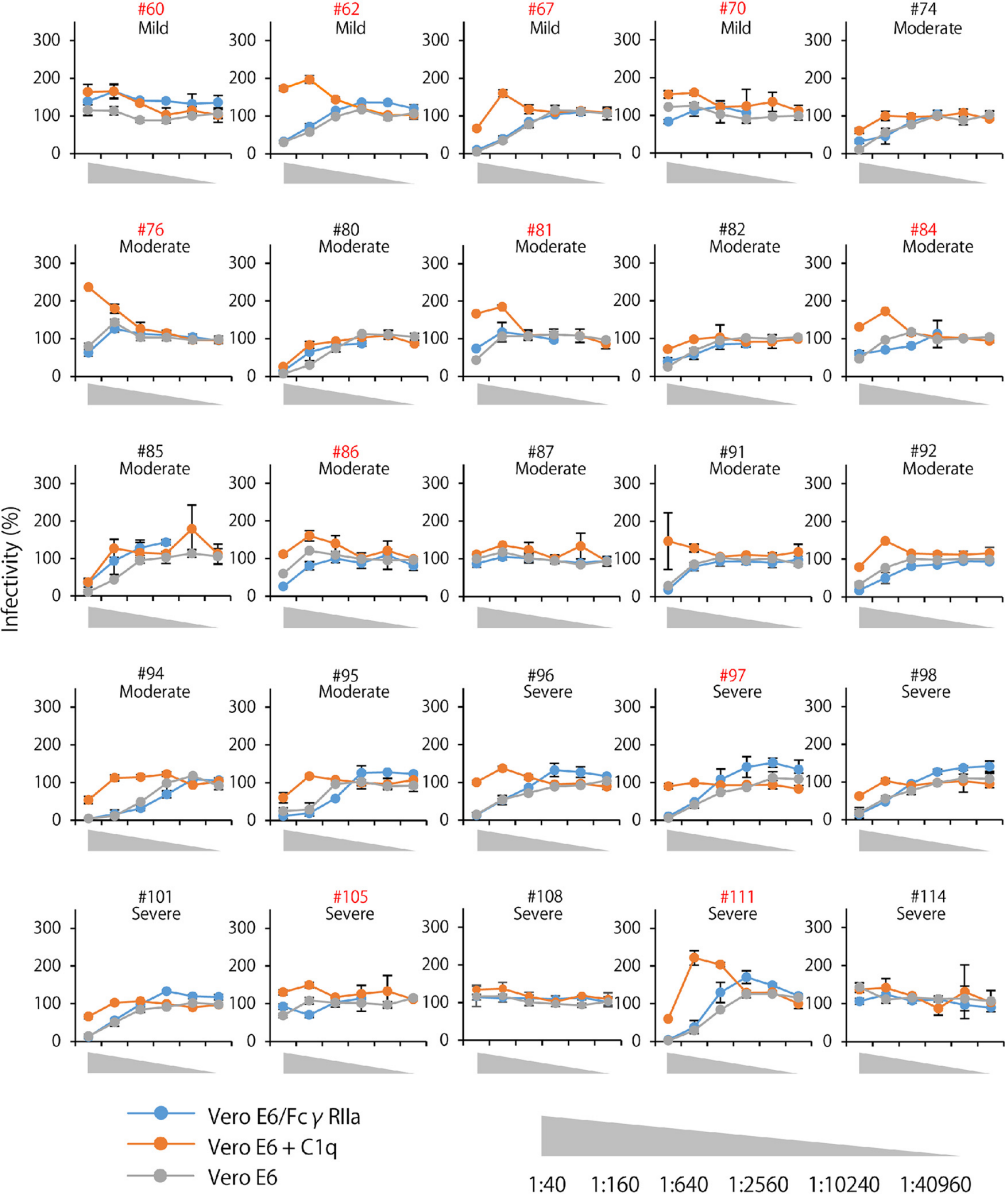
Neutralization and ADE activities found in serum samples against pseudotyped virus. Fifty representative serum samples from COVID-19 convalescents (numbers 26–46), acute patients with mild (numbers 48, 51, 56, 57, 60, 62, 67, and 70), moderate (numbers 74, 76, 80–82, 84–87, 91, 92, 94, and 95), and severe (numbers 96–98, 101, 105, 108, 111, and 114) symptoms were investigated. The relative numbers of infected cells were calculated by setting the number of GFP-positive cells in the absence of the serum to 100%. Each experiment was done twice and results are shown as means and standard deviations. ADE-positive sample numbers are shown in red.

To investigate FcγR-mediated ADE, we established Vero E6 cells expressing FcγRIIa (Vero E6/FcγRIIa cells) and confirmed that a previously reported ADE antibody specific to EBOV (ZGP12/1.1) (28) indeed increased the infectivity of VSV pseudotyped with EBOV glycoprotein (GP) (VSV-EBOV) in this cell line (Fig. 2A). Similarly, C1q-mediated ADE was confirmed using ZGP12/1.1 and Vero E6 cells lacking FcγR (29) (Fig. 2B). This antibody showed neither neutralizing nor ADE activity in the absence of FcγR or C1q (Fig. 2C). Using these assay conditions, we investigated FcγR- and C1q-mediated ADE activities in the serum samples collected for this study (Fig. 3). Vero E6/FcγRIIa cells were infected with VSV-SARS2 mixed with serially diluted serum samples (1:40 to 1:40960) for the FcγR-mediated ADE assay. For the C1q-mediated ADE assay, Vero E6 cells were infected with the virus mixed with serially diluted serum samples in the presence of C1q. As an ADE-negative-control assay (i.e., neutralization condition), Vero E6 cells were infected with the virus mixed with the serum dilutions in the absence of C1q.

ADE assays were conducted for 50 representative serum samples whose OD values were more than 1.0 in ELISA, out of 56 samples that were considered to be SARS-CoV-2 IgG-positive (Fig. 1 and Table 1). Infectious titers that were increased 150% or more compared to virus alone (i.e., without serum) were defined as ADE (Fig. 3 and Table 2). Of the 21 serum samples from the convalescents, 12 (numbers 29, 33, 34, 35, 36, 37, 41, 42, 43, 44, 45, and 46) sera had increased virus infectivity at either of the dilutions when FcγR was expressed on the cell surface (Vero E6/FcγRIIa cells). Twelve sera (numbers 29, 32, 33, 34, 35, 36, 37, 41, 43, 44, 45, and 46) had increased virus infectivity when C1q was present in the culture medium (Vero E6 + C1q). Overall, 61.9% (13/21) of the convalescent serum samples showed FcγR- and/or C1q-mediated ADE activities. Among the COVID-19 acute patients’ sera, FcγR-mediated ADE activity was found in 1 (number 60), 1 (number 85), and 2 (numbers 97 and 111) samples from the patients with mild (*n* = 8), moderate (*n* = 13), and severe (*n* = 8) symptoms, respectively. C1q-mediated ADE was detected at a slightly higher rate in the patients, with 4 (numbers 60, 62, 67, and 70), 5 (numbers 76, 81, 84, 85, and 86), and 2 (numbers 105 and 111) samples from those with mild, moderate, and severe symptoms, respectively. Overall, ADE activities were found in 41.4% (12/29; 4/8, 5/13, and 3/8 from mild, moderate, and severe groups, respectively) of the sera from these acute patients but there was no significant difference in the ADE-positive rates among the groups (Table 2). When Vero E6 cells were infected in the absence of FcγR or C1q, none of the samples induced ADE but most of them showed neutralizing activity (Table 1 and Fig. 3). Overall, we found that 50% (25/50) of the SARS-CoV-2 IgG-positive sera had ADE antibodies (Table 2). It was also important to note that although the infectivity was not increased more than 150%, almost all the other sera had decreased neutralizing activities in the presence of FcγR and/or C1q, suggesting a counteracting effect by ADE antibodies (i.e., neutralization was seemingly weakened by ADE). Particularly, some samples (numbers 30, 31, 38, 48, 91, 92, and 96) were very marginal (more than 140% but less than 150%) with respect to the ADE cutoff value.

**TABLE 2:** ADE-positive rates in IgG-positive sera of COVID-19 acute and convalescent patients

Group	FcγR	C1q	FcγR and C1q	FcγR and/or C1q
Convalescent	57.1% (12/21)	57.1% (12/21)	52.4% (11/21)	61.9 (13/21)
Mild disease	12.5% (1/8)	50% (4/8)	12.5% (1/8)	50.0% (4/8)
Moderate disease	7.7% (1/13)	38.5% (5/13)	7.7% (1/13)	38.5% (5/13)
Severe disease	25% (2/8)	25% (2/8)	12.5% (1/8)	37.5 (3/8)
All acute patients	13.8% (4/29)	37.9% (11/29)	10.3% (3/29)	41.4 (12/29)
Total	32% (16/50)	46% (23/50)	28% (14/50)	50% (25/50)

### Antibody concentrations determining neutralizing or ADE activity.

**FIG 4:**
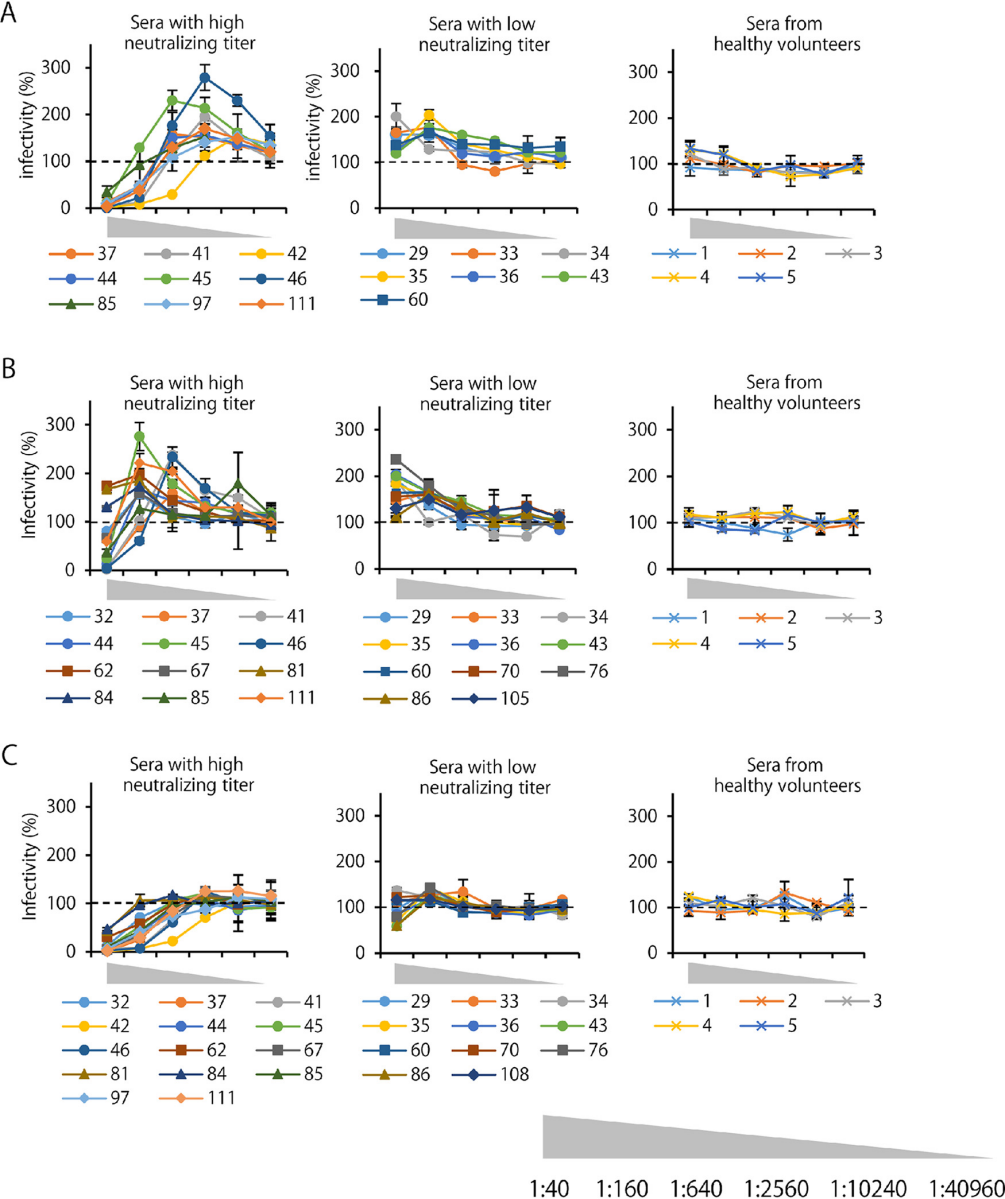
Comparison of optimal ADE conditions between serum samples with high and low neutralizing activities. Data shown in Fig. 3 are reconstituted for the comparison of FcγR-mediated ADE (A), C1q-mediated ADE (B), and neutralization (C) curves of each sample. The ADE-positive serum samples are divided into 2 groups based on their neutralizing titers; high (1:320–1:2560) and low (<1:160). Serum samples from convalescents, patients with mild, moderate, and severe symptoms are shown by circles, squares, triangles, and diamonds, respectively.

Next, we focused on serum dilutions (i.e., antibody concentrations) that gave the optimal conditions for ADE. They indeed varied depending on the sample, but we were able to identify 2 groups based on the difference in their neutralizing titers (Fig. 4). In most of the sera that showed comparatively high neutralization titers (1:320–1:2560), neutralizing activity was prominent even in the presence of FcγR and C1q at lower dilutions (i.e., higher antibody concentrations) of the samples and the peak ADE activity was observed at the dilution that gave subneutralizing conditions (1:640–1:10240 and 1:160–1:2560 dilutions in FcγR- and C1q-mediated ADE assays, respectively) (Fig. 4A and B, left panels). In contrast, none of the sera with lower neutralizing titers (<1:160) showed neutralizing activity even at the lowest dilution of the samples (1:40) under ADE conditions, and these samples had increased virus infectivity most efficiently at comparatively low dilutions (1:40 or 1:160) in both FcγR- and C1q-mediated ADE assays (Fig. 4A and B, middle panels). In the non-ADE condition (i.e., infection in Vero E6 cells without FcγR and C1q), all of the former samples showed dose-dependent neutralizing activities, whereas most of the latter samples showed no or weak neutralizing activity even at the lowest dilution (1:40) of the samples (Fig. 4C).

### Correlation between IgM levels and C1q-mediated ADE.

**FIG 5:**
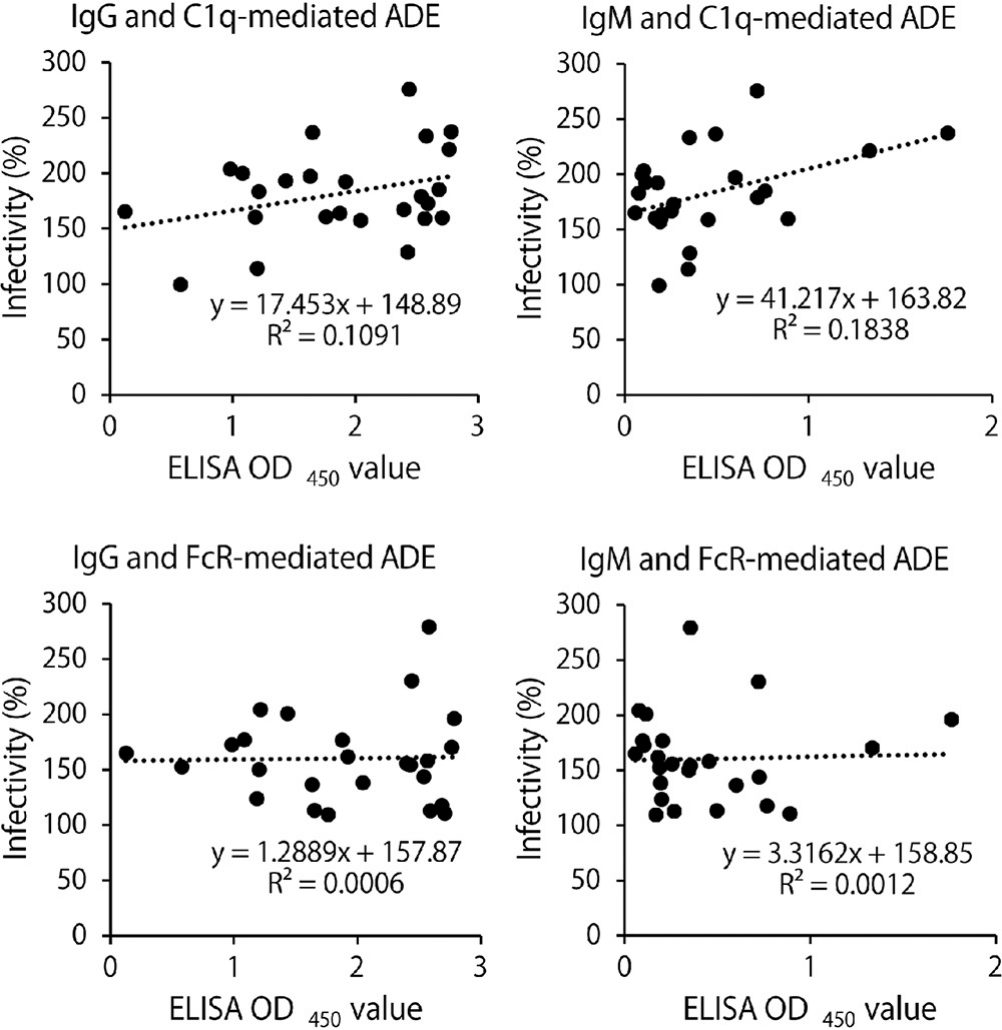
Correlation between IgG/IgM antibody levels and ADE activities. Serum samples that showed FcR- and/or C1q-mediated ADE activities (numbers 29, 32, 33, 34, 35, 36, 37, 41, 42, 43, 44, 45, 46, 60, 62, 67, 70, 76, 81, 84, 85, 86, 97, 105, and 111) were analyzed. ELISA OD values and peak relative infectivity (%) were obtained from the data shown in Fig. 1 and 3, respectively.

We then investigated correlations between antibody levels and ADE activities (Fig. 5). The ELISA OD values of IgG and IgM in the serum samples that showed FcR- and/or C1q-mediated ADE activities were analyzed with their peak ADE activities (i.e., peak percentages of relative infectivity shown in Fig. 3). We found a weak positive correlation between IgG levels and C1q-mediated ADE (R^2^ = 0.1091). It was noted that higher correlation (R^2^ = 0.1838) was found between IgM levels and C1q-mediated ADE. In contrast, there was no appreciable correlations for FcR-mediated ADE. These results suggest an important role of IgM for C1q-mediated ADE.

### ADE in multiple cycles of replication of SARS-CoV-2.

**FIG 6:**
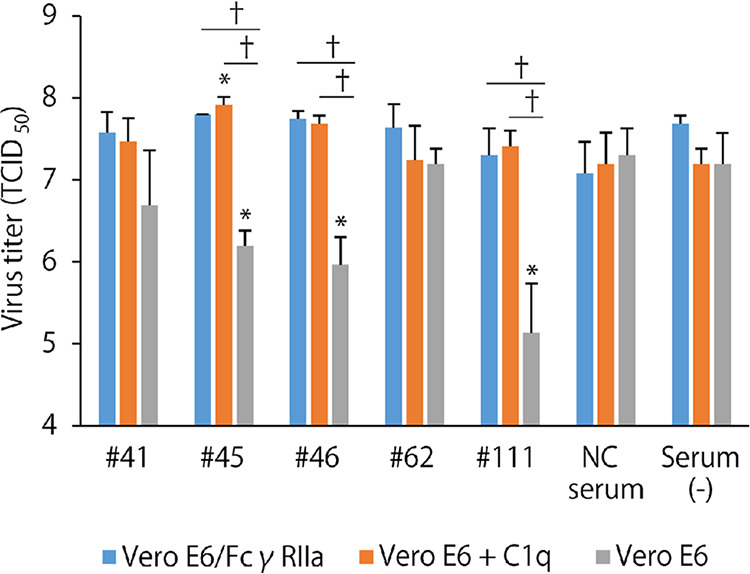
ADE and reduced neutralization of SARS-CoV-2 by serum samples of COVID-19 patients. SARS-CoV-2 strain JPN/TY/WK-521 was inoculated into Vero E6 and Vero E6/FcγRIIa cells and grown in the presence of serum samples of numbers 41, 45, 46, 62, 111, and a negative control (NC) diluted at 1280, 320, 1280, 320, 640, and 320, respectively. For C1q-mediated ADE, the virus was grown in Vero E6 cells with the sera and the medium supplemented with C1q during the incubation. The data are shown as means and standard deviations of three independent experiments. Significant differences (*P* < 0.05) compared to the serum (-) in the respective conditions (i.e., Vero E6/FcγRIIa, Vero E6 + C1q, or Vero E6) were determined using one-way ANOVA followed by Dunnett's multiple-comparison test and are shown with asterisks. Daggers indicate significant differences (*P* < 0.05) between the 2 indicated groups determined using Student's *t* test.

Finally, ADE activities of the representative samples (numbers 41, 45, 46, 62, and 111) that showed more than 200% enhancement were tested in multiple cycles of replication using infectious SARS-CoV-2 strain JPN/TY/WK-521 (Fig. 6). The virus was grown in the presence or absence of FcγR and C1q with the sera diluted to subneutralizing conditions (i.e., 1:320–1:1280 dilution depending on the sample). Three (numbers 45, 46, and 111) of the five tested sera showed significant neutralizing activity when Vero E6 cells were infected in the absence of FcγR and C1q. In contrast, the neutralizing activity of the sera was completely abolished when the virus was propagated in Vero E6/FcγRIIa and in Vero E6 cells with soluble C1q in the culture medium. More importantly, one of the sera (number 45) had significantly increased virus infectivity compared to the serum (-) control when the virus replicated in Vero E6 cells in the presence of C1q. These data clearly demonstrated that the COVID-19 patients’ sera had ADE potential for SARS-CoV-2 infection *in vitro* and also indicated that ADE antibodies had the ability to counteract neutralizing activity in the presence of FcγR on the target cell surface or C1q in the culture medium.

## DISCUSSION

The potential ADE risk associated with SARS-CoV-2 infection is a global concern from the aspect of disease exacerbation by reinfection and vaccine-induced preexisting immunity (30, 31). However, it is still controversial whether ADE of SARS-CoV-2 infection is involved in the immunopathological effects affecting clinical outcomes. It has also been suggested that FcγR-mediated infectivity enhancement, which is the most commonly accepted ADE mechanism, plays a limited role since FcγR is not expressed on the major target cells for SARS-CoV-2 (8). The present study demonstrates that SARS-CoV-2 infection induces antibodies that can cause ADE through another mechanism, namely, C1q-mediated ADE, which may be more likely to occur in respiratory epithelial cells, which SARS-CoV-2 primarily infects.

In our study, the C1q-mediated ADE activity, like the FcγR-mediated ADE, was detected in more than half of the COVID-19 convalescent-phase sera. C1q-mediated ADE has been proposed for HIV, EBOV, Marburg virus, and human parvovirus (23, 24, 32, 33). This mechanism relies on C1q molecules bridging the virus-antibody complexes and C1q receptors on cell surfaces, leading to increased viral attachment to target cells; the activation of the complement classical pathway is unlikely to be required (23, 28, 29). Since C1q is abundantly present in the plasma and C1q receptors are expressed ubiquitously (34), C1q-mediated ADE may potentially occur in a wide variety of cell types. We assume that it is not essential to trigger C1q expression for the phenomenon since the C1q concentration (50 μg/mL) we used in our experiments was equivalent or rather lower than that in normal human plasma. Interestingly, a recent study demonstrated that complement components, including C1q are deposited in the capillaries of the interalveolar septa and on alveolar epithelial cells in COVID-19 patients (35), suggesting the likelihood of C1q-mediated ADE in the respiratory tissue of COVID-19 patients. Furthermore, other studies reported that secondary infection with SARS-CoV-2 induced more severe symptoms than the primary infection in a few clinical cases (36, 37). These observations may imply the *in vivo* relevance of ADE in SARS-CoV-2 infection, whereas the association between the presence of ADE antibodies and severity of secondary infection still needs to be clarified by detailed serological and clinical studies.

Notably, C1q- and/or FcγR-mediated ADE activities were also observed in 41.4% of the sera collected from acute patients (median, 6; interquartile range [IQR], 2.25–9 days after onset), suggesting the potential of ADE to promote virus replication even in the acute phase of primary SARS-CoV-2 infection. Interestingly, C1q-mediated ADE tended to be more prominent than FcγR-mediated ADE in the patients with mild and moderate symptoms (numbers 47–95), compared to the convalescent-phase sera (numbers 26–46) (Table 2). Since C1q binds more efficiently to IgM and polymeric IgM-like IgG than to monomeric IgG antibodies (38, 39), we assume that some anti-S-specific IgM antibodies may contribute to disease exacerbation if ADE occurs *in vivo*. In this study, not only IgG but also IgM antibodies were detected in some of the acute COVID-19 patients although we could not identify a correlation between the presence of ADE antibodies and disease severity (Table 2). It should also be noted that severely symptomatic patients were mostly the elderly which might have a negative impact on immune response and antibody production. Further studies with larger numbers of clinical samples with different age groups are needed to investigate the possible roles of C1q-mediated ADE in COVID-19 patients.

Previous studies showed that epitopes recognized by ADE antibodies were generally distinct from those for neutralizing antibodies while some ADE antibodies show neutralizing activity at high concentrations (19, 40–42). Interestingly, a study reported that ADE antibodies to MERS-CoV recognizing an epitope on the receptor binding site of the trimeric S stabilized the receptor binding site, which triggered a conformational change of MERS-CoV S, leading to enhanced infection (17). A more recent study demonstrated that some antibodies recognizing the N-terminal domain of the SARS-CoV-2 S induce a conformational change of the receptor binding domain and enhance the viral infectivity independently from FcγR- and C1q-mediated ADE pathways (43). However, we assume that such ADE antibodies are not predominantly induced by natural infection by SARS-CoV-2 since none of the sera tested in the present study had increased the viral infectivity in the absence of FcγR and C1q.

Previous studies on other viruses have indicated that ADE is often observed at subneutralizing concentrations of ADE antibodies (11, 41). Accordingly, in our FcγR- and C1q-mediated ADE assays, antibody concentrations (i.e., serum dilutions) that gave optimal ADE activities were different among the samples and it depended on the neutralizing activity of the sample (Fig. 4). Our data suggest that neutralizing activity is dominant when overall levels of SARS-CoV-2-specific antibodies are high, and the ADE risk may appear in a period when neutralizing antibodies are decreased below the level of detection. Although previous studies suggested a positive correlation between the disease severity and IgG response, which might support the production of ADE antibodies (5, 44, 45), it could be assumed that such a correlation is simply due to the difference in the magnitude of virus infection and the subsequent immune response, but may not be associated with the presence of ADE antibodies.

While convalescent plasma therapies have been tested for SARS, MERS, 2009 H1N1 pandemic influenza, and EBOV disease (46–49), this approach has been clinically applied to COVID-19 patients and promising results have been obtained (50–52). Our study demonstrated that neutralizing antibodies were detectable in most of the tested convalescent-phase sera collected at 28–73 days after disease onset (median, 47; IQR, 38.5–50.5), whereas more than half of them showed either FcγRIIa- or C1q-mediated ADE activity. Considering the presence of antibodies that potentially enhance SARS-CoV-2 infection, ADE may raise a potential issue for passive immunization with COVID-19 convalescent plasma, as well as its therapeutic utility against SARS-CoV-2 variants (53–56). For the therapeutic use of COVID-19 convalescent plasma, both neutralizing and ADE activities should be investigated carefully to confirm that neutralizing activity is not negated by ADE antibodies at a concentration for clinical application.

In this study, we showed multiple routes of ADE of SARS-CoV-2 infection *in vitro* using serum samples collected from COVID-19 acute and convalescent patients. It remains to be investigated whether vaccine-induced antibodies also have the potential to cause ADE. Although the clinical impact of SARS-CoV-2 ADE needs to be further investigated, our data suggest the importance of careful monitoring of the antibody properties in convalescent and vaccinated individuals.

### Limitation of the study.

The main limitation of the study is that the sample size is not large enough to generalize the prevalence of ADE antibodies and the differences among the groups. This is a concern since only 50 samples were used for the neutralization and ADE analyses, including comparisons among several different groups (i.e., severity of the disease, ages of patients and days after onset) with different characteristics (i.e., neutralization, FcR-mediated, and C1q-mediated ADE).

## MATERIALS AND METHODS

### Cells.

African green monkey kidney Vero E6 cells, Vero E6 cells expressing the type II transmembrane serine protease (Vero-TMPRSS2) (57), and Vero E6/FcγRIIa cells were cultured in Dulbecco’s minimum essential medium (DMEM; Sigma-Aldrich, St. Louis, MO, USA) supplemented with 10% fetal bovine serum (FBS; Gibco, Waltham, MA, USA), 100 U/mL penicillin, and 0.1 mg/mL streptomycin (Gibco) at 37°C in 5% CO_2_. Vero E6/FcγRIIa cells were generated as described previously (58). Briefly, a retrovirus carrying the FcγRIIa gene was generated by cotransfecting human embryonic kidney 293T cell-derived Platinum-GP cells (Cell Biolabs, San Diego, CA, USA) with the plasmids, pMXs-puro encoding the FcγRIIa gene and pCAGGS encoding the VSV glycoprotein (G), using TransIT-LT1 (Mirus, Madison, WI, USA). The collected supernatant was then inoculated into Vero E6 cells. Transduced cells stably expressing FcγRIIa were selected with the growth medium containing 3 μg/mL puromycin (Sigma-Aldrich) and cloned by limiting dilution. Vero E6/FcγRIIa cells were maintained in the presence of 3 μg/mL puromycin (Sigma-Aldrich). In the serological assays, the cells were maintained in DMEM containing 2% FBS (2%-FBS/DMEM) after virus inoculation.

### Viruses.

SARS-CoV-2 strain JPN/TY/WK-521 (59) was propagated in Vero-TMPRSS2 cells maintained in 2%-FBS/DMEM at 37°C in 5% CO_2_. Infectious titers of SARS-CoV-2 were determined in 50% tissue culture infectious dose (TCID_50_) assays using Vero-TMPRSS2 cells. Using VSV containing the green fluorescent protein (GFP) gene instead of the VSV G gene, VSV-SARS2 and VSV-EBOV were generated as described previously (60, 61). Pseudotyped VSVs were pretreated with a neutralizing MAb to VSV G (VSV-G [N] 1-9) (33) to abolish the background infectivity of parental VSV. Pseudotyped VSVs were inoculated into each cell line cultured on 96-well plates, and infectious units (IU) were determined 20 h later by counting the number of GFP-expressing cells using IN Cell Analyzer 2500HS (GE Healthcare).

### Serum samples.

A total of 112 serum samples (23 healthy volunteers, 21 convalescents, and 68 acute patients with laboratory [PCR]-confirmed COVID-19) were used in this study (see Table S1 in the supplemental material). The healthy volunteers did not have a history of SARS-CoV-2 infection and were unvaccinated and asymptomatic. The 68 patients (34 male and 34 female) were referred to and hospitalized at the National Hospital Organization (NHO) Hokkaido Medical Center from February 15, 2020, to January 22, 2021. The COVID-19 patients’ ages ranged from 21 to 101 years old with median of 73. Based on their symptoms, the patients were divided into three groups; mild (*n* = 25; numbers 47–71), moderate (*n* = 24; numbers 72–95), and severe (*n* = 19; numbers 96–114) symptomatic cases. The severity of the COVID-19 infection was defined by Clinical Management of Patients with COVID-19 with mild cases constituting those without need of oxygen administration, moderate cases as those requiring oxygen, and severe cases as those requiring mechanical ventilation. The convalescent-phase serum samples (numbers 26–46) were collected from other individuals (*n* = 21; 5 male and 16 female) whose symptoms had been categorized with the same definition as mild (*n* = 15), moderate (*n* = 5), and severe (*n* = 1) and 23 healthy volunteers (3 male and 20 female). The median ages were 37 years (IQR, 24–46) for healthy volunteers, 41 (IQR, 25.5–74.5) for convalescents, 53 (IQR, 39.5–82) for mildly symptomatic patients, 83 (IQR, 70.25–90.75) for moderately symptomatic patients, and 74 (IQR, 61–89) for severely symptomatic patients. Days from the onset of the disease to blood sampling were 0–24 (median, 6; IQR, 2.25–9) and 28–73 (median, 47; IQR, 38.5–50.5) for the acute and convalescent patients, respectively.

### ELISA.

The supernatant of Vero-TMPRSS2 cells infected with JPN/TY/WK-521 was filtered with a 0.45 μm pore membrane (Sartorius, Goettingen, Germany) and ultracentrifuged (28,000 rpm in a Beckman SW32 rotor [Beckman Coulter Brea, CA, USA], 2 h) with a sucrose cushion (15% sucrose in PBS) to concentrate the virus particles. Then the pellet was resuspended with a disruption buffer (0.05 M Tris-HCl [pH 7.6], 0.5% Triton X-100, 0.6 M KCl). The disrupted virus particles were diluted at 20 μg/mL with PBS and used as an ELISA antigen. ELISA plates (Nunc Maxisorp; Invitrogen, Carlsbad, CA, USA) were coated with the viral antigen and blocked with 3% skim milk (Becton, Dickinson, Franklin Lakes, NJ, USA) in PBS. Serum samples diluted at 1:100 in PBS containing 1% skim milk and 0.05% Tween 20 were plated in duplicate, and the bound antibody was detected using horseradish peroxidase (HRP)-conjugated goat anti-human IgG (H+L) (Jackson Immuno Research, West Grove, PA, USA) and HRP-conjugated donkey anti-human IgM Fc_5μ_ (Jackson Immuno Research). The reaction was visualized by adding 3,3′,5,5′-tetramethylbenzidine (TMB; Sigma-Aldrich) and optical density (OD) at 450 nm was measured. Serum samples of healthy volunteers were used to calculate the cutoff OD value (average ± 3 SD).

### Neutralization assay.

Neutralization assays were performed as described previously (28, 29, 42). Serum samples were serially diluted (4-fold or 2-fold dilutions) with 2%-FBS/DMEM. The diluted sera were mixed with VSV-SARS2 (200-300 IU/0.1 mL), incubated for 1 h, and inoculated into Vero-TMPRSS2 or Vero E6 cells plated in 96-well plates. Each neutralization titer was determined as the highest dilution that gave 50% inhibition of the numbers of GFP-positive cells.

### ADE assays.

ADE assays were performed as described previously (28, 29, 42). Vero E6 and Vero E6/FcγRIIa cells cultured in 96-well plates were used for C1q- and FcγR-mediated ADE assays, respectively. To detect FcγR-mediated ADE, VSV-SARS2 (200–300 IU/0.1 mL) was mixed with serially diluted serum samples (4-fold dilutions), incubated for 1 h, and inoculated into Vero E6/FcγRIIa cells. For C1q-mediated ADE assays, the virus was mixed and incubated with the serial dilutions of the serum samples and purified C1q (50 μg/mL; Sigma-Aldrich) before inoculating into the Vero E6 cells. Infected cells expressing GFP were counted 20 h later using IN Cell Analyzer 2500HS (GE Healthcare, Atlanta, GA, USA). The relative number of infected cells was calculated by setting the GFP count in the absence of serum samples to 100%, and relative infectivity that showed more than 150%, which was nearly equal to the values of average + 3 standard deviations given by healthy volunteer control sera, were defined as ADE. For the validation of ADE assays, VSV-EBOV and an anti-EBOV GP monoclonal antibody, MAb ZGP12/1.1, which possesses ADE activity for EBOV infection (28), were used.

To test ADE in authentic SARS-CoV-2 infection, JPN/TY/WK-521 (10^4^ TCID_50_/mL) was mixed with equal volume of appropriately diluted serum samples (1:160–1:640 in 2%-FBS/DMEM), incubated for 1 h, and 0.1 mL of the mixture was inoculated into Vero E6 and Vero E6/FcγRIIa cells on 24-well plates. For C1q-mediated ADE assays, the medium was supplemented with C1q (final concentration of 50 μg/mL). The inoculum was removed and 0.5 mL of 2%-FBS/DMEM containing each serum sample (final dilution of 1:320–1:1280) was added. For C1q-mediated ADE assays, the infected cells were maintained in the medium supplemented with C1q (50 μg/mL). Supernatants were collected 48 h after infection and virus titers were determined as TCID_50_.

### Statistical analyses.

Data were analyzed using GraphPad Prism version 8 for Windows (GraphPad Software, San Diego, CA, USA). Kruskal-Wallis test followed by Dunn’s multiple-comparison test and the Student's *t* test were used to analyze each data set as indicated in the figure legends.

### Ethical statement.

Serum samples (one per patient) were collected with informed consent from each patient at NHO Hokkaido Medical Center and this study was approved by the institutional ethical committees in NHO Hokkaido Medical Center (number 2020-12-5) and Hokkaido University (number 2020-6).
